# Dietary Acculturation and Food Habit Changes among Chinese Immigrants in Portugal

**DOI:** 10.3390/nu15081886

**Published:** 2023-04-14

**Authors:** Catarina Li, Elisabete Carolino, Joana Sousa

**Affiliations:** 1 Laboratório de Nutrição, Faculdade de Medicina, Centro Académico de Medicina de Lisboa, Universidade de Lisboa, 1649-028 Lisboa, Portugal; 2 H&TRC—Health & Technology Research Center, ESTeSL—Escola Superior de Tecnologia da Saúde, Instituto Politécnico de Lisboa, 1990-096 Lisboa, Portugal; 3 Instituto de Saúde Ambiental, Faculdade de Medicina, Universidade de Lisboa, 1649-028 Lisboa, Portugal

**Keywords:** dietary acculturation, Chinese immigrants, food habits, Portugal

## Abstract

Chinese individuals who immigrate to a Western country tend to change their eating patterns and behaviors depending on how long they live in the host country. This is dietary acculturation, and it can have a positive or negative impact on eating habits. Thus, we aimed to characterize the dietary acculturation of the Chinese immigrant community in Portugal and check the trend of the direction of this acculturation. The study involved 213 immigrants and assessed food consumption, meal patterns, and dietary acculturation. A mean Western acculturation score of 70.1 ± 8.9. was identified and 71.4% had a high Western acculturation score. No one had low or very high Western acculturation. Participants who have a higher level of acculturation tend to have higher energy and fat intake. The likelihood of mixing meals, including, and combining, Chinese and Portuguese meals and foods is associated with time spent in Portugal. Efforts should be made to encourage Chinese immigrants to make a positive dietary transition during their acculturation process.

## 1. Introduction

Chinese immigrants living in Western countries have a higher prevalence of chronic diseases, such as type 2 diabetes mellitus (T2DM), cardiovascular disease (CVD), and obesity. Although the reasons for this high prevalence are not completely understood, dietary acculturation is proposed as a contributing factor [1]. Dietary acculturation refers to the process that occurs when members of a minority group adopt the eating patterns/food choices of the host country [2]. This process is multidimensional, dynamic, complex, and varies considerably depending on personal, cultural, and environmental factors, and can have a positive or negative impact on eating habits. This means that it is not a simple process in which a person moves linearly from one end of the continuum of acculturation (traditional) to the other (acculturated) [3]. After people migrate to a new environment, dietary acculturation is considered as one of the behavioral consequences. Over this course, immigrants can keep their traditional food pattern, adopt the food pattern of the host country (excluding the intake of traditional food), or maintain certain eating habits from the country of origin while incorporating food from the new country of residence [2].

Several studies have examined the influence or consequences of dietary acculturation on food intake and dietary patterns [4,5,6,7]. Research supports the reality that Chinese immigrants living in Western countries may have an increased intake of processed foods high in sugar, fat, and salt, which may contribute to a negative health status. A longitudinal survey carried out in the United States revealed that acculturation increases with length of residence in the country, and is accompanied by an increase in dietary energy density, as well as an increase in the percentage of fat and sugar intake [4]. Similar results were obtained from studies that involved Chinese Canadians, as they also showed similar occurrences. Unfavorable dietary changes after immigration included increased sugary beverage intake, increased food portion sizes, increased consumption of convenience products, processed, and energy-dense food [5]. From the aforementioned evidence, immigrants who are approaching Western culture and simultaneously changing their eating patterns to the Western style in a negative way are more likely to develop chronic diseases [8].

As immigrants move to a new host country with a strong cultural heritage, they may change their lifestyle to the dominant culture, resulting in a rapid change in dietary patterns [8]. There are significant differences between Chinese and Portuguese food cultures, namely regarding the concept of food, the way to serve food, the timing and composition of meals, among other things [9,10,11]. Food in Western countries is considered part of a rational diet, placing nutrition first instead of the colors, shapes, and flavors of the food, while in China, flavor prevails more, probably due the scarcity and hunger that the population experienced in the past [9]. At meal times, eating individual and separate dishes on square tables is common in Portugal, but, in Chinese culture, the sharing system is used in most situations, highlighting values such as unity and harmony through the use of round tables [9,11]. Regarding meal composition, Portuguese meals consist of three main meals, adding usually a morning and an afternoon snack, while the Chinese generally have only three meals a day (breakfast, lunch, and dinner) [10]. Furthermore, Portuguese meal times tend to be later than in Chinese customs [10]. All these factors could contribute to the changes in eating patterns of the Chinese immigrants.

To date, a few current studies have explored changes in eating habits and their relationship with acculturation among Chinese immigrants. Therefore, the aim of this study is to characterize the changes in eating habits of the Chinese immigrant community in Portugal, and to identify the existence of a shift towards a Westernized acculturation of this population.

## 2. Materials and Methods

### 2.1. Study Design

A quantitative ethnographic study was conducted in Lisbon (capital of Portugal) to better understand the complex and dynamic processes of the dietary changes of Chinese immigrants. The study protocol was approved by the Ethics Committee of the Academic Center of Medicine of Lisbon (project ID: No. 83/22).

### 2.2. Data Collection

A questionnaire was applied in the form of a semi-structured interview by the researcher to the study participants, about 15–20 min long, at their workplace. Before the interview, each participant completed a survey to provide demographic data including age, gender, marital status, length of residence, among other brief information to characterize the participants’ context in the research study. The dietary acculturation model proposed by Satia-Abouta et al. was used to guide the interview questions [12].

The participants were recruited through Chinese commercial establishments (e.g., clothing stores, restaurants, grocery stores, as well as Chinese community institutions and educational institutions) around the Lisbon zone. If the participant wished to be interviewed at another time of the day (for example, outside of their working hours), the researcher would return at that previously scheduled time to increase the receptivity of the sample. All survey instruments were presented in Portuguese with a Chinese translation shown on the back page. As inclusion criteria, researchers included Chinese immigrants between 18 and 64 years old who immigrated to Portugal for at least 1 year and were able to communicate in Mandarin, Chinese, or Portuguese. Data were collected between 10 April 2022 and 1 June 2022.

### 2.3. Measures

#### 2.3.1. Dietary Assessment

Diet was measured using 24 h recall, which consists of an interview with the participant to fully record daily food consumption from the previous day in order to characterize current eating habits [13]. The food quantification manual, plastic food models, and standard measuring cups were also used to estimate portion sizes. During the interviews, the author also asked participants for some descriptive information about their meal patterns and the main foods consumed at each meal.

Data were recorded in a Microsoft Excel database and calculated using the existing foods in the Food Composition Table of the National Health Institute Doctor Ricardo Jorge (INSA) version 5.0 2021 [14], and for Chinese foods we used the Food Composition Table of China 2019 published by the Institute of Nutrition and Food Safety, China CDC, to complement the nutrient values of these foods [15].

#### 2.3.2. Dietary Acculturation

This part of the questionnaire was adapted from a previously validated scale developed by Satia-Abouta et al. [12] and resulted in 22 questions that compared eating/cultural behaviors after immigration, in relation to habits in the country of origin, and involved two categories: Eastern acculturation and Western acculturation, with 11 questions for each one. Each category included a list of items that reflected the foods and behaviors of either the traditional Chinese diet (Eastern acculturation) or the Westernization of the dietary pattern (Western acculturation). These options were randomly dispersed without specific mention in relation to the type of acculturation. The items were further scored so that summing up the total score for each participant resulted in four levels of acculturation: low, moderate, high, or very high. If the participant obtained a “low” score, it represented low Western acculturation and preserved Eastern eating habits. If the participant achieved a “very high” score, they were fully acculturated to Western eating habits. 

The details about the treatment of data regarding dietary acculturation are explained in Appendix A.

### 2.4. Data Analysis

The collected data were analyzed using the IBM Statistical Package for Social Science software, New York, NY, USA (version 27.0). Descriptive statistics were used to characterize the sample under study (frequency analysis (*n*, %)) for qualitative data, and mean and standard deviation for quantitative data. To test the normality of the data, tests for adjustment to the normal distribution were used, relying on the Kolmogorov–Smirnov tests for samples with dimensions greater than 50. To compare two independent groups, the *t*-test was used for two independent samples. To compare the intake of macronutrients and micronutrients with the values of the DRIs, the *t*-test was used for one sample. For the comparison of two or more independent groups, the ANOVA test at 1 fixed factor was used, after confirming normality and verifying the homogeneity of variances. The results were considered significant at the 5% significance level.

### 2.5. Ethical Considerations

The study was approved by the Ethics Committee of the Academic Medical Center of Lisbon (project ID: No. 83/22). All participants received information and conditions regarding the objective of the study, both oral and written. All participants included in the study signed the informed consent document, and all data collected were anonymized to guarantee the ethical and legal aspects.

## 3. Results 

### 3.1. Demographic Characteristics 

A total of 213 participants were interviewed. The sample consisted of 117 (54.9%) Chinese immigrant women with a mean age of 36 ± 11 years (range 18 to 64 years). 

The average residence time in Portugal was 15 ± 7 years, varying between 1 and 40 years. The households consisted of an average of 4 ± 2 members, and it should be noted that the largest number of family members in one household was 10. Almost 80% (166 (78.7%)) lived with family members, followed by 125 (59.2%) with a partner, 18 (8.5%) alone, and only 6 (2.8%) with friends. All participants were from mainland China. The origin of the respondents was diverse, highlighting that the vast majority came from the province of ZheJiang (75.6%), located in the south of China, as reported in other studies about Chinese immigrants in Portugal [16,17]. The predominant levels of education were high school (38.0%) and elementary school (25.8%). Regarding marital status, 130 (61.0%) respondents were married, and 73 (34.3%) were single. Regarding professional categories, a great diversity of careers can be seen in this group, where being a merchant was dominant (67.6%). 

In relation to the Portuguese language domain, 116 (54.5%) reported “Insufficiency” and 202 (95.3%) reported having Mandarin as their language preference.

See Table 1 for the full demographic results.

### 3.2. Dietary Assessment

Table 2 presents the average nutritional intake for each nutrient analyzed and the respective DRIs based on gender. For both genders, there was an average intake below the recommended amount in terms of energy, AGMU, carbohydrates, fiber, vitamin A, vitamin C, vitamin D, thiamine, riboflavin, folate, potassium, calcium, magnesium, iron, and zinc. The nutrients with an average consumption higher than recommended were proteins, simple sugars, vitamin E, vitamin B12, sodium, and phosphorus in both genders, and the consumption of lipids in females. 

### 3.3. Meal Patterns 

The average number of main meals consumed prior to immigration was three meals per day (breakfast, lunch, and dinner). The consumption of intermediate meals (mid-morning and afternoon snacks) was rarely observed. After arrival in Portugal, this trend was maintained. 

It was observed that the breakfast foods consumed were markedly Western style, characterized by the inclusion of milk, yogurt, coffee, cereal, toast, and bread/croissants with cheese and/or ham. On the other hand, the frequency of consumption of typically Chinese foods such as porridge, noodles, and steamed buns was low. The participants’ lunch was mostly a typical Chinese lunch characterized by rice as the main source of carbohydrates, and various meat, fish, and vegetable dishes. Only the participants who frequented restaurants had Western meals. Dinner, the meal most often characterized as a family meal, was mainly composed of the typical Chinese dinner, often with rice or noodles as the main source of carbohydrates, along with several different dishes. The main cooking methods mentioned were sautéed and stewed. On the other hand, some of the participants reported frequently consuming Western-style dinners, including foods such as pizza, hamburgers, and French fries, among others. 

Regarding the time available for meals, it was observed that the immigrants in the present study tended to follow Western customs, characterized by having meals later when compared to the routines of Chinese society. Additionally, it was observed that there was a high integration of Portuguese meals in the diet of this population after immigration, where 53.3% of the participants reported having a combination of traditional Portuguese meals and traditional Chinese meals. It should be noted that even so, 46.2% of the subjects preferred to maintain the habit of having traditional Chinese meals and only 0.5% (one participant) reported having the habit of having more traditional Portuguese meals in their daily lives (Table 3).

### 3.4. Dietary Acculturation 

A total of 213 participants had valid results for the dietary acculturation score.

Table 4 shows that the average Western acculturation score was 70.1 ± 8.9. The lowest score in the study was 43 and the highest score was 96. It was found that 28.6% of the participants had a moderate Western acculturation score and 71.4% a high Western acculturation score. It is also noteworthy that there was no record of subjects with a low Western acculturation or a very high Western acculturation.

By associating acculturation with energy intake, there is statistical significance between both (*p* < 0.05), where participants who have a higher level of acculturation tend to have a higher energy intake. The same is confirmed for fat intake, when correlating the variables (*p* < 0.05), where participants who have a higher level of acculturation tend to have a higher fat intake (Table 5). There was no statistical correlation between overall demographic characteristics and acculturation. 

Finally, it can also be seen that the longer the residence time in Portugal, the greater the probability of mixing meals, including, and combining, Chinese and Portuguese meals and foods (*p* < 0.05).

## 4. Discussion

This study aimed to further understand the changes in eating habits among Chinese immigrants in Portugal, and identified the existence of a shift towards a Westernized acculturation in this population.

Previous studies have shown that Asians who migrate to Western countries tend to adopt a Western diet, resulting in reduced intake of fiber and increased intake of energy, lipids, and sugars [18]. This occurrence was observed in the Chinese immigrant population of this study. The main dietary trends after immigration are reflected in increased intake of protein, lipids, and simple sugars, and reduced fiber intake. 

The results of the present study seem to indicate that there is a transition in the energy structure from the three main macronutrients, in which the percentage of energy intake obtained from carbohydrates is gradually replaced by the energy contribution from lipids, accompanied by a slight increase in the intake of proteins as an energy source [19]. One of the explanations for this fact is that the Chinese population used to eat large amounts of rice, noodles, as well as other carbohydrate-rich sources in the three main meals when they were in China. After immigrating, the amount of carbohydrates declined, apparently explained by the fact that it was replaced by foods rich in animal protein, typical of the Western diet.. These data are also supported by a longitudinal study that examined the eating behaviors of Chinese immigrants settled in Australia, which concluded that as this population increased their consumption of protein and lipids, their consumption of carbohydrates decreased significantly [20].

When analyzing the nutritional intake for micronutrients, it was found that this was lower than the DRIs for vitamin A, vitamin C, vitamin D, thiamine, riboflavin, folate, potassium, calcium, magnesium, iron, and zinc, which is in agreement with similar studies [19,21]. These intake deficits may be related to several factors such as the decline in the intake of cereals, dairy products, vegetables, and fruits. Furthermore, some traditional Chinese food lacked nutritional information in the qualifying sources used; consequently, it could be undervalued regarding the intake of certain micronutrients. On the contrary, regarding sodium intake, it is observed that both males and females have a very high intake, which corresponds to 7.2 g salt/day and 7.6 g salt/day, respectively, and can be related to the addition of various sauces to daily cooking, beyond table salt (such as soy sauce and oyster sauce), as observed in the qualitative analysis of the 24 h recall.

From this perspective, the decline in the nutritional quality of the diet is a worrying reality, since Chinese immigrants may become more susceptible to diet-related health problems similar to those that affect the general population in Europe, such as obesity, cardiovascular diseases, and diabetes [8,22,23]. Studies carried out with Chinese immigrants from other countries identified the need for culturally sensitive and linguistically appropriate resources of dietary guidelines for the immigrant population [7,24].

Regarding meal patterns, comparing pre-immigration and post-immigration meals, the patterns tended to remain the same in these immigrants. The main meals included breakfast, lunch, and dinner, and this custom was maintained after arriving in Portugal. Even so, breakfast is where the great Westernization of eating habits can be noted, as reported in other countries such as Spain, the United States, and Australia [1,25,26]. Participants in the present study reported preferring to have a markedly Western breakfast instead of the typical Eastern one as it is more practical and easier to prepare. Furthermore, the preparation of some characteristic Chinese foods requires, beyond time and family support, specific culinary skills. For example, a typical Chinese breakfast food, rice porridge, requires a cooking time of at least 30 to 40 min. As most of the participants in the present study were merchants (67.6%) (following the trend of the Chinese immigrant population in Portugal [27]), a lack of time was one of the most frequent reasons reported when asked about neglecting a Chinese breakfast because their lifestyle was hurried. Therefore, it can be assumed that the choice for a Western-style breakfast is based on convenience rather than taste preference. These data are consistent with the study carried out by Satia et al., in which they observed that the first meal to be Westernized was breakfast among Chinese American women, but most of the interviewees reported having Chinese-style lunches and dinners [28]. It can be seen, then, that Chinese immigrants try to maintain their traditional eating patterns while incorporating the eating habits of the new host country into their diet [1].

Dietary acculturation has been used to investigate the process by which groups of immigrants adopt the dietary patterns of the host country [2]. However, several factors can affect the food choices, eating patterns, and lifestyles of immigrants. Immigration and the loss of some eating habits from the country of origin to adopt the habits of the host country means having a higher level of dietary acculturation. Lower acculturation reflects, on the contrary, that behavior is less open to the new culture, maintaining customs similar to those of the place of origin, and therefore not changing eating habits or behaviors [28]. It is observed that the immigrants in the present study have a high level of acculturation and integration into Portuguese society, with 71.4% showing a high Western acculturation level. Moreover, the longer the Chinese immigrants reside in Portugal, the more they prefer to mix Chinese and Portuguese meals. From this fact it can be said that the trend is to incorporate increasingly Western-style meals into their daily diet [26]. This may reflect, at the same time, a positive and negative impact on the Chinese immigrant population residing in Portugal. On the one hand, this process has favored the incorporation of some healthy and typical food of the Mediterranean diet, as observed in the 24 h recall, such as olive oil, salads, and some aromatic herbs. However, this has also resulted in some unhealthy changes, such as the consumption of fast food (the greatest contributor to the increase in energy and fat intake in the diet) and also the preference for potentially less healthy eating habits, for example by increasing food portion sizes and dining out in Western-style restaurants [29]. From this perspective, efforts must be made to ensure that healthy Western foods are adopted throughout the acculturation process, since the biggest challenge found in this immigrant population results in the adoption of unhealthy eating habits, leading to a consequent decline in future health status.

As a proposal for future investigations, longitudinal studies are required to explore whether there is indeed a causal relationship between changes in dietary patterns and the existence of dietary acculturation among Chinese immigrants in Portugal.

### 4.1. Strength and Limitations

This study has some limitations. First, a convenience sample was used, which compromises the extrapolation of data to the Chinese community in Portugal. Secondly, so far, there are no validated assessment instruments to meet the objectives of the study. All instruments for assessing dietary intake have inherent weaknesses. Assessing the food intake of this population was a challenge, as it involved several Chinese foods and quantities that were difficult to estimate. On the other hand, when calculating nutritional intake, some foods lacked nutritional information in relation to micronutrients in the qualifying sources used, and therefore another limitation was the undervaluation of the actual intake of certain micronutrients. 

The strengths of the current study include the in-depth interviews with the participants, providing qualitative insights into the experience of dietary acculturation for this reserved cultural group. Furthermore, interviews were conducted by a bilingual language skills researcher, allowing interviews to be conducted in the participant’s preferred language. In addition, efforts were made to minimize possible biases during the interviews by trying to increase the accuracy of the 24-hour recall data collection using the photographic food quantification manual, food templates, and standard measuring cups to estimate portion sizes. This obtained a better identification of the products consumed and their quantities to consider the most correct nutritional information adjusted to the actual consumption.

### 4.2. Implications

The present study has some clinical implications that should be considered. The current research identified that Chinese immigrants are potentially at risk of nutritional imbalance. To encourage Chinese immigrants to make a positive dietary transition during the acculturation process, it is necessary to target interventions to promote positive health behaviors in this population. An effort will be emerging on the part of researchers and public health policymakers to formulate clear and culturally sensitive policies to facilitate the planning of specific nutritional interventions for this ethnic population, such as encouraging the retention of existing healthy habits, and/or the adoption of new healthy behaviors. This decision contributes both to more effective nutritional education programs and helps health professionals throughout their clinical practice, ensuring access to culturally appropriate care and optimized health outcomes for this population.

## 5. Conclusions

This study states that Chinese immigrants experience the process of dietary acculturation after arriving in Portugal, where almost ¾ of the participants experienced a high level of Western acculturation. It was observed that the higher the level of dietary acculturation in these participants, the higher the energy density and fat intake in the diet. The pattern of pre- and post-immigration meals was not affected at all, with participants mainly maintaining the three daily meals: breakfast, lunch, and dinner. Breakfast was the meal where the Westernization of eating habits was markedly denoted. Moreover, the participants of the present study appeared to experience a negative nutritional transition from the adopted dietary pattern. Cereals and low-fat dishes have been replaced by a Westernized diet high in fat and low in carbohydrates, resulting in an insufficient intake of grains and cereals, dairy products, vegetables, and fruits, and an excessive consumption of fat and salt. Efforts should be made to encourage Chinese immigrants to make a positive dietary transition during the acculturation process.

## Figures and Tables

**Table 1:** Demographic characteristics of participants.

		Total
*n*		213
Age (years), mean (±SD)		36 ± 11 years
Years residing in Portugal, mean (±SD)		15 ± 7 years
Sex, *n* (%)	Female	117 (54.9%)
	Male	96 (45.1%)
Place of birth, *n* (%)	China, ZheJiang	161 (75.6%)
	China, ShanDong	15 (7.0%)
	China, FuJian	9 (4.2%)
	China, other places	28 (13.2%)
Level of education, *n* (%)	Elementary school	73 (34.3%)
	High school	81 (38.0%)
	University degree or higher	59 (27.7%)
Marital Status, *n* (%)	Single	73 (34.3%)
	Married/Partner	130 (61.0%)
	Divorced	7 (3.3%)
	Widowed	3 (1.4%)
Employment status, *n* (%)	Merchant	144 (67.6%)
	Other careers	41 (19.3%)
	Student	15 (7.0%)
	Unemployed	9 (4.2%)
	Retired	4 (1.9%)
Portuguese proficiency	Unsatisfactory	116 (54.5%)
	Satisfactory	52 (24.4%)
	Very good	32 (15.0%)
	Excellent	13 (6.1%)
Language preference	Mandarin	202 (95.3%)
	Portuguese	10 (4.7%)
	Other	0 (0.0%)

**Table 2:** Relationship between the average dietary pattern and the DRIs by gender.

	Male			Female		
	Mean (±SD)	DRI’s	*p*	Mean (±SD)	DRI’s	*p*
Energy (kcal/d)	1564.0 ± 411	2100	0.00	1435.8 ± 321.4	1750	0.00
Protein (g/d)	70.7 ± 19.6	65	0.01	61.9 ± 16.7	55	0.00
Lipids (g/d)	68.1 ± 19.3	47–70 *	0.00	65.0 ± 17.4	39–58 *	0.00
SFA (g/d)	15.5 ± 6.1	16	0.44	14.9 ± 5.7	14	0.07
MUFA (g/d)	22.1 ± 8.7	30	0.00	21.2 ± 8.7	25	0.00
PUFA (g/d)	20.9 ± 8.3	23	0.02	18.6 ± 8.1	19	0.58
Carbohydrates (g/d)	158.7 ± 58.8	263–341 *	0.00	148.9 ± 43.5	219–284 *	0.00
Simple sugar (g/d)	27.6 ± 23.9	<23	0.06	26.6 ± 16.9	<19	0.00
Fiber (g/d)	10.7 ± 4.4	25–30	0.00	11.3 ± 4.9	25–30	0.00
Vitamin A (µg/d)	440.7 ± 271.6	800	0.00	479.4 ± 429.3	700	0.00
Vitamin C (mg/d)	85.2 ± 80.6	100	0.08	76.8 ± 61.2	100	0.00
Vitamin D (µg/d)	3.9 ± 3.9	10	0.00	3.4 ± 3.1	10	0.00
Vitamin E (mg/d)	17.1 ± 7.5	14	0.00	15.4 ± 7.7	14	0.05
Thiamin (mg/d)	0.9 ± 0.3	1.4	0.00	0.9 ± 0.4	1.2	0.00
Riboflavin (mg/d)	1.0 ± 0.4	1.4	0.00	0.9 ± 0.5	1.2	0.00
Niacin (mg/d)	14.6 ± 5.7	14	0.32	10.8 ± 4.8	12	0.01
Vitamin B6 (mg/d)	1.6 ± 0.5	1.6	0.26	1.4 ± 0.5	1.6	0.00
Vitamin B12 (µg/d)	4.2 ± 6.8	2.4	0.01	4.2 ± 9.5	2.4	0.05
Folate (µg/d)	175.5 ± 63.7	400	0.00	171.0 ± 78.8	400	0.00
Sodium (mg/d)	2890.1 ± 865.7	1900	0.00	3041.8 ± 855.1	1900	0.00
Potassium (mg/d)	1885 ± 595.6	3600	0.00	1686.8 ± 538.3	3600	0.00
Calcium (mg/d)	358.1 ± 183.8	1000	0.00	384.4 ± 249.7	1000	0.00
Phosphorus (mg/d)	875.8 ± 242.9	720	0.00	759.0 ± 271.9	720	0.12
Magnesium (mg/d)	191.7 ± 71.8	330	0.00	177.6 ± 69.0	330	0.00
Iron (mg/d)	7.1 ± 3.6	12	0.00	6.9 ± 4.3	12	0.00
Zinc (mg/d)	6.8 ± 2.6	12.5	0.00	5.9 ± 2.4	7.5	0.00

* Average value of the reference interval of the DRIs.

**Table 3:** Main meals served.

	% (*n*)
Traditional Chinese meals	46.2 (98)
Traditional Portuguese meals	0.5 (1)
Combination of both	53.3% (113)
Others	0% (0)

**Table 4:** Western Acculturation Score.

	Mean (±SD)	% (*n*)	Min–Max
Acculturation	70.1 (±8.9)		43.0–96.0
Western acculturation	Low	0.0% (0)	
	Moderate	28.6% (61)	
	High	71.4% (152)	
	Very high	0.0% (0)	

**Table 5:** Acculturation and Energy and Fat intake.

		Moderate Acculturation	High Acculturation	Value *p*
Energy intake	Mean (±SD)	1389.6 (±379.4)	1535.3 (±357.9)	
	% (*n*)	28.6% (61)	71.4% (152)	0.009
Fat intake	Mean (±SD)	62.3 (± 20,2)	68.1 (± 17,3)	
	% (*n*)	28.6% (61)	71.4% (152)	0.035

## Data Availability

The data presented in this study are available on request from the corresponding author.

## Appendix A

### Dietary Acculturation

All questions were based on the 5-point Likert-scale (0 = “Not applicable”, 1 = “Never”, 2 = “Rarely”, 3 = “Sometimes”, 4 = “Often” and 5 = “Always”).

For the treatment of data regarding dietary acculturation, a score was developed according to the response to 22 questions (11 questions were about Eastern acculturation and 11 questions were about Western acculturation). The options were coded by “0”, “1”, “2”, “3”, “4” and “5” according to the level of Western acculturation. The higher the number, the greater the Western acculturation. For example, “Using cutlery at meals” is considered a symbol of Western acculturation; if the answer was “Always”, it would be coded as “5”, which means more Western acculturation. In the case of questions referring to Eastern customs, the scores were proportionally inverted. For example, “Using chopsticks at meals” is considered a symbol of Eastern acculturation, and if the answer were “Always”, this option would already be coded as “1”, which means less Western acculturation, and so on.

The global scale could range from 22 (if all responses are scored “1”) to 110 (if all responses are scored “5”). The sum of everyone’s total score was calculated and designated as the “Western acculturation score”. The score was divided into four intervals and the classification was made using the theoretical values of the scale, with its final interpretation being made using the following scale:

<32.999—Low western acculturation

33–65.999—Moderate western acculturation

66–98.999—High western acculturation

>99—Very high western acculturation
